# Internal control for real-time polymerase chain reaction based on MS2 bacteriophage for RNA viruses diagnostics

**DOI:** 10.1590/0074-02760160380

**Published:** 2017-04-06

**Authors:** Miriam Ribas Zambenedetti, Daniela Parada Pavoni, Andreia Cristine Dallabona, Alejandro Correa Dominguez, Celina de Oliveira Poersch, Stenio Perdigão Fragoso, Marco Aurélio Krieger

**Affiliations:** 1 Fundação Oswaldo CruzFundação Oswaldo CruzInstituto Carlos ChagasLaboratório de GenômicaCuritibaPRBrasilFundação Oswaldo Cruz-Fiocruz, Instituto Carlos Chagas, Laboratório de Genômica, Curitiba, PR, Brasil; 2 Universidade Federal do ParanáUniversidade Federal do ParanáDepartamento de Bioprocessos e BiotecnologiaCuritibaPRBrasilUniversidade Federal do Paraná, Departamento de Bioprocessos e Biotecnologia, Curitiba, PR, Brasil; 3 Fundação Oswaldo CruzFundação Oswaldo CruzInstituto de Biologia Molecular do ParanáCuritibaPRBrasilFundação Oswaldo Cruz-Fiocruz, Instituto de Biologia Molecular do Paraná, Curitiba, PR, Brasil

**Keywords:** diagnostics, Real-time PCR, internal control, HCV, RNA viruses

## Abstract

**BACKGROUND:**

Real-time reverse transcription polymerase chain reaction (RT-PCR) is routinely used to detect viral infections. In Brazil, it is mandatory the use of nucleic acid tests to detect hepatitis C virus (HCV), hepatitis B virus and human immunodeficiency virus in blood banks because of the immunological window. The use of an internal control (IC) is necessary to differentiate the true negative results from those consequent from a failure in some step of the nucleic acid test.

**OBJECTIVES:**

The aim of this study was the construction of virus-modified particles, based on MS2 bacteriophage, to be used as IC for the diagnosis of RNA viruses.

**METHODS:**

The MS2 genome was cloned into the pET47b(+) plasmid, generating pET47b(+)-MS2. MS2-like particles were produced through the synthesis of MS2 RNA genome by T7 RNA polymerase. These particles were used as non-competitive IC in assays for RNA virus diagnostics. In addition, a competitive control for HCV diagnosis was developed by cloning a mutated HCV sequence into the MS2 replicase gene of pET47b(+)-MS2, which produces a non-propagating MS2 particle. The utility of MS2-like particles as IC was evaluated in a one-step format multiplex real-time RT-PCR for HCV detection.

**FINDINGS:**

We demonstrated that both competitive and non-competitive IC could be successfully used to monitor the HCV amplification performance, including the extraction, reverse transcription, amplification and detection steps, without compromising the detection of samples with low target concentrations. In conclusion, MS2-like particles generated by this strategy proved to be useful IC for RNA virus diagnosis, with advantage that they are produced by a low cost protocol. An attractive feature of this system is that it allows the construction of a multicontrol by the insertion of sequences from more than one pathogen, increasing its applicability for diagnosing different RNA viruses.

Polymerase chain reaction (PCR) has been used extensively as a diagnostic tool since the early 1990s. The use of PCR has revolutionised the diagnostic detection of microorganisms by providing rapid, specific and sensitive detection of microbe’s nucleic acid in a variety of samples.

Initially, false-positive results, a consequence of reaction contamination with previously generated amplicons, were the main concern of those who were using this new technique. Measures were adopted to avoid contamination with pre-amplified nucleic acids and substantial improvement achieved with the introduction of real-time PCR. This is because real-time PCR is a closed tube system which does not require post-PCR analysis of amplicons; still considered a major source of contamination and false-positive results when executing this technique.

Additionally, there has been an increased focus on minimising the impact of false-negative results; i.e., assuring that negative results truly represent absence of PCR diagnostic targets. This is because false-negative results can occur from failure of one of the test steps (nucleic acid extraction, reverse transcription reaction, PCR set up or amplification).

The first measures to control false-negative results were based on the addition of exogenous nucleic acids to the PCR reaction, usually a plasmid, so that the presence of any inhibitory substance in the samples would also affect the amplification of the exogenous material. Single and multiple internal controls based on plasmids were constructed for using in diagnostics of several pathogens (Stöcher et al. 2003, Dingle et al. 2004, Hymas et al. 2005, Maaroufi et al. 2006).

This type of control, however, only assessed the amplification step. In the case of viral detection, it is necessary to control prior steps as well, such as viral particles recovery from plasma and viral nucleic acid extraction. Therefore, strategies were developed to overcome this limitation. Initially, the exogenous nucleic acid was added before nucleic acid extraction, which controlled this step but did not properly assess the relative loss of viral particles during this process. Hence, the ideal control should be a protected nucleic acid that is subjected to exactly the same steps as a viral particle. In addition, the construction of a protected DNA or RNA would shield the nucleic acid from degradation by nucleases or hydrolysis after long-term storage, which is particular important if one needs to use RNA as a control.

Viruses, or pseudoviral particles, containing the same kind of nucleic acid as the target (DNA or RNA), in a stable structure, which are designed to undergo simultaneously the same steps as the target pathogen, have been used in the detection assays of viral targets (Cleland et al. 1999, Garson et al. 2005, Clancy et al. 2008). Non-pathogenic viruses to humans and animals are preferred over pathogenic ones for security reasons, and viruses that are easily and cheaply produced are preferred (Dreier et al. 2005, Gerriets et al. 2008, Ninove et al. 2011).

Two classes of exogenous internal controls have been used in real-time PCR assays. The competitive control has a sequence specific to the target microorganism that allows the use of the same primer pair in the PCR reaction, but uses a discriminatory probe. The non-competitive control uses a detection sequence unrelated to the microorganism to be detected. The competitive control can mimic the amplification kinetics of the target sequence; however, it can compete with the target sequence at low pathogen loads. Besides, for each target, a competitive control must be constructed. The non-competitive control can be associated with several targets; nevertheless, the optimisation of a multiplex PCR with two different primer pairs can be difficult, and the control amplification kinetics often do not reflect those of the target amplification (Rosenstraus et al. 1998, Hoorfar et al. 2004, Sharma et al. 2014). The benefits and disadvantages of the strategies above should be carefully analysed in each situation when choosing between them.

Several reports have been published on the use of non-competitive controls in viral detection, using viruses such as bovine diarrhea virus (Cleland et al. 1999), phocine distemper virus (Clancy et al. 2008), murine cytomegalovirus (Garson et al. 2005) and the bacteriophages T4 and MS2 (Dreier et al. 2005, Rolfe et al. 2007, Gerriets et al. 2008, Ninove et al. 2011), which have been used in diagnosis of several DNA and RNA viruses, including hepatitis C virus (HCV).

Competitive controls were developed to several viruses based on bacteriophages lambda and Qβ (Stöcher et al. 2003, Stöcher & Berg 2004, Villanova et al. 2007, Meng et al. 2009). Pseudoviral particles comprising a viral coat protein encapsulating a nucleic acid specific to a pathogen have been developed, being the first one a commercial initiative called “Armored RNA” based on MS2 coat protein (Pasloske et al. 1998). After that, several works reported the use of this kind of competitive control (WalkerPeach et al. 1999, Drosten et al. 2001, Beld et al. 2004, Cheng et al. 2006, Zhao et al. 2007, Wei et al. 2008, Meng et al. 2009, Zhan et al. 2009, Felder & Wölfel 2014, Sharma et al. 2014).

In this paper, we describe the construction of a virus-modified particle, based on MS2 bacteriophage, to be used as a competitive internal control for the diagnosis of RNA viruses. We demonstrated its applicability for HCV diagnosis. This internal control allows us to monitor the extraction, reverse transcription, amplification and detection steps. This particle fulfills the characteristics of an ideal internal control; i.e., it is produced by an economic protocol, it is not infectious to animals, it does not possess any sequence present in clinical samples and it is ribonuclease resistant. Additionally, its propagation by plasmid replication using the bacterial DNA polymerase is less prone to mutations and the modification of viral polymerase gene makes it impossible for replication by infection to occur, protecting the bacterial stocks from contamination.

## MATERIALS AND METHODS

*Internal control construction* - Complementary DNA from the MS2 RNA (Roche, Mannheim, Germany) was synthesized using the primer CLONMS2-R (Table I) and ImProm II reverse transcriptase (Promega, Madison, WI, USA). The primers forward CLONMS2-F and reverse CLONMS2-R were used to amplify a 3,569 bp region of the genome using GoTaq^TM^ DNA polymerase (Promega). The amplicon was purified from an agarose gel and inserted into the pGEM-T Easy vector (Promega), generating the pGEM-T Easy-MS2 plasmid. pGEM-T Easy-MS2 was subsequently digested with *Not*I, generating a fragment of 3,604 bp containing the MS2 genome, which was purified and filled in using the Klenow fragment of DNA polymerase (New England Biolabs, Herts, United Kingdom).

**TABLE I:** Oligonucleotides used for internal control (IC) construction and for real time polymerase chain reaction

Oligonucleotide^*^	Sequence (5’-3’)	Position^†^
CLONMS2-F	GGGTGGGACCCCTTTCG	1-17
CLONMS2-R	TGGGTGGTAACTAGCCAAG	3,551-3,569
MS2-F	TCCTAAAAGATGGAAACCCGATT	1,669-1,691
MS2-R	GGCCGGCGTCTATTAGTAGATG	1,739-1,718
MS2-P	VIC-CCTCAGCAATCGCAGCAAACTCCG-MGBNFQ^§^	1,693-1,716
HCV-F^‡^	CGGGAGAGCCATAGTGGT	130-147
mHCV-P	VIC-CGTGGACTGACACGCGAGACT-MGBNFQ	149-169

*: F = forward; R = reverse; P = probe. ^†^: position on the MS2 genome is based on GenBank accession number NC_001417.1; position on the hepatitis C virus (HCV) genome is based on GenBank reference sequence AF009606.1. ^‡^: oligonucleotides from Duarte et al. (2010), with modifications. ^§^: MGB = minor groove binding; NFQ = non-fluorescent quencher. Oligonucleotides HCV-R1, HCV-R2, HCV-P, HIV-F, HIV-R and HIV-P are components of the NAT HIV/HCV kit (Patent number PI0600715-5/INPI/Brazil). HCV-P and HIV-P have a FAM moiety at their 5′ ends.

pET-47b(+) (Novagen, Darmstadt, Germany) was digested with *Xba*I and *Xho*I enzymes (New England Biolabs) liberating a 255 bp fragment of the polylinker region. After purification, the ends were filled in by the Klenow fragment of DNA polymerase I and dephosphorylated with shrimp alkaline phosphatase (Amersham Pharmacia Biotech, Piscataway, NJ, USA). The 3,604 bp region originated from the pGEM-T Easy-MS2 excision was ligated to pET-47b(+), generating pET-47b(+)-MS2.

**Fig. 1:**
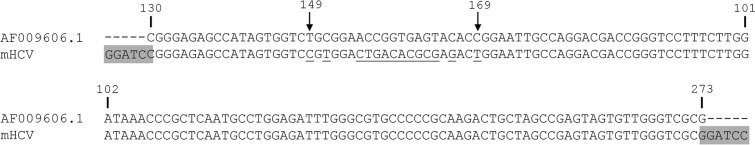
: modified hepatitis C virus (mHCV) sequence aligned to HCV subtype 1a reference sequence: numbers indicating nucleotide positions refer to HCV reference sequence AF00906.01. Arrows indicate the first and last probe positions. Underlined, in the probe region, there are the bases in the mHCV sequence that differ from HCV genotype 1a reference sequence. *Bam*HI sites are in gray.

A pUC57-mHCV (m stands for mutated), with an inserted sequence of 144 nt derived from HCV subtype 1a polyprotein, reference sequence AF009606.1, position 130 to 273 cloned into the *Bam*HI site, was purchased from GenScript (Piscataway, NJ, USA). The mHCV sequence has altered bases compared with the reference sequence from position 149 to 169 (Fig. 1).

pET-47b(+)-MS2 and pUC5-mHCV were digested with *Bam*HI (New England Biolabs). The former was linearised and the second released the mutated HCV sequence, which was ligated using T4 DNA ligase (Invitrogen, Carlsbad, CA, USA) into the linearised vector at position 2,057 of the MS2 genome (based on GenBank accession number NC_001417.1), generating pET47b(+)-MS2-mHCV.

The MS2 regions in plasmids pET-47b(+)-MS2 and pET-47b(+)-MS2-mHCV were sequenced using internal oligonucleotides (not shown).

Plasmids propagation was conducted after transformation into *Escherichia coli* strains Top10F’ (for pGEM-T Easy-MS2 and pET-47b(+)-MS2) and DH5α (pUC57-mHCV and pET-47b(+)-MS2-mHCV). Plasmid minipreps were obtained using a QIAprep Spin Miniprep kit or a HiSpeed Plasmid Midi kit (Qiagen, Düren, Germany) and after restriction enzyme digestion, the fragments were purified from the agarose gel using a High Pure PCR Product Purification kit (Roche, Mannheim, Germany). Confirmatory PCRs to check the presence of inserts and their orientation were also performed (not shown) and selected clones were sequenced at Macrogen Inc. (Seoul, South Korea).

*Internal control particles production* - pET-47b(+)-MS2 and pET-47b(+)-MS2-mHCV were transformed in *E. coli* BL21 and transcription of the MS2 genome was induced by the addition of 1 mM isopropyl-1-thio-β-D-galactoside. After 3 h, the bacterial cells were pelleted by centrifugation at 16,000 × g. The bacteriophages particles in the supernatant were precipitated with 10% (w/v) polyethylene glycol for 18 h at 4ºC and centrifuged at 150,000 × g for 2 h. The pelleted viral particles were then suspended in SM medium (0.1 M NaCl, 8 mM MgSO_4_, 0.05 M Tris-HCl pH 7.5, 0.01% gelatin).

The titer of MS2-like particles derived from pET-47b(+)-MS2 (to be used as the non-competitive internal control) was determined as plaque forming units (PFU)/mL) by plating assays on *E. coli* XL1-Blue F’. MS2-mHCV particles derived from pET-47b(+)-MS2-mHCV (to be used as the competitive internal control) were quantified by real-time PCR, using the MS2-like particles as a reference, and the titer was expressed as MS2 PFU-equivalent (PFU-eq).

*Samples* - Quantified HCV RNA samples were purchased from AcroMetrix (HCV OptiQuant HCV RNA/Life Technologies, Carlsbad, CA, USA) and HCV RNA Linearity Panel PHW804 (SeraCare Life Sciences, Milford, CT, USA). An HIV clinical sample was kindly provided by LACEN/PR/Brazil.

*RNA extraction* - RNA from HCV clinical samples and bacteriophage particles was extracted using BioPur (Biometrix, Curitiba, Brazil) and IBMP extraction kits (IBMP, Curitiba, Brazil) from 200 mL of plasma sample, bacteriophage solution or a mixture of the two, according to the manufacturer’s instructions. An automatised solution also was tested using BioRobot® MDx and a QIAamp Media MDx kit from 600 mL of material (Qiagen). The purified RNA was eluted with 40-100 mL of appropriated buffer.

*One-step real-time PCR* - The MS2-like and MS2-mHCV internal controls (ICs) were amplified using 400 nM of primers (MS2-F/MS2-R or HCV-F/HCV-R) and 200 nM of probe (MS2-P or mHCV-P). The multiplex HCV/MS2-like IC was done with 400 nM of primers MS2-F, MS2-R, HCV-F and HCV-R1, 58 nM HCV-R2 and 450 nM of probes (MS2-P and HCV-P). The multiplex reaction HCV/MS2-mHCV IC was done with 400 nM of primers HCV-F and HCV-R1, 58 nM HCV-R2 and 450 nM of probes (HCV-P and mHCV-P). The multiplex reaction HIV/MS2-like IC was amplified with 400 nM of the MS2 primers, 400 nM HIV-F and HIV-R primers and 450 nM of probes (MS2-P and HIV-P). All the reactions were performed in a 25 mL reaction with 10 mL of RNA, 1× PCR amplification buffer with Taq DNA polymerase and dNTPs (IBMP, Curitiba, Brazil) and 0.1 U of reverse transcriptase (IBMP, Curitiba, Brazil). All the oligonucleotide sequences are shown in Table I and in reference (Duarte et al. 2010).

For all amplifications, the thermal cycle conditions began with an initial step at 51ºC (30 min) and 95ºC (10 min); followed by 60 cycles at 95ºC (30 s), 60ºC (1 min), in an ABI 7500 Thermal Cycler (Life Technologies).

## RESULTS

**Fig. 2:**
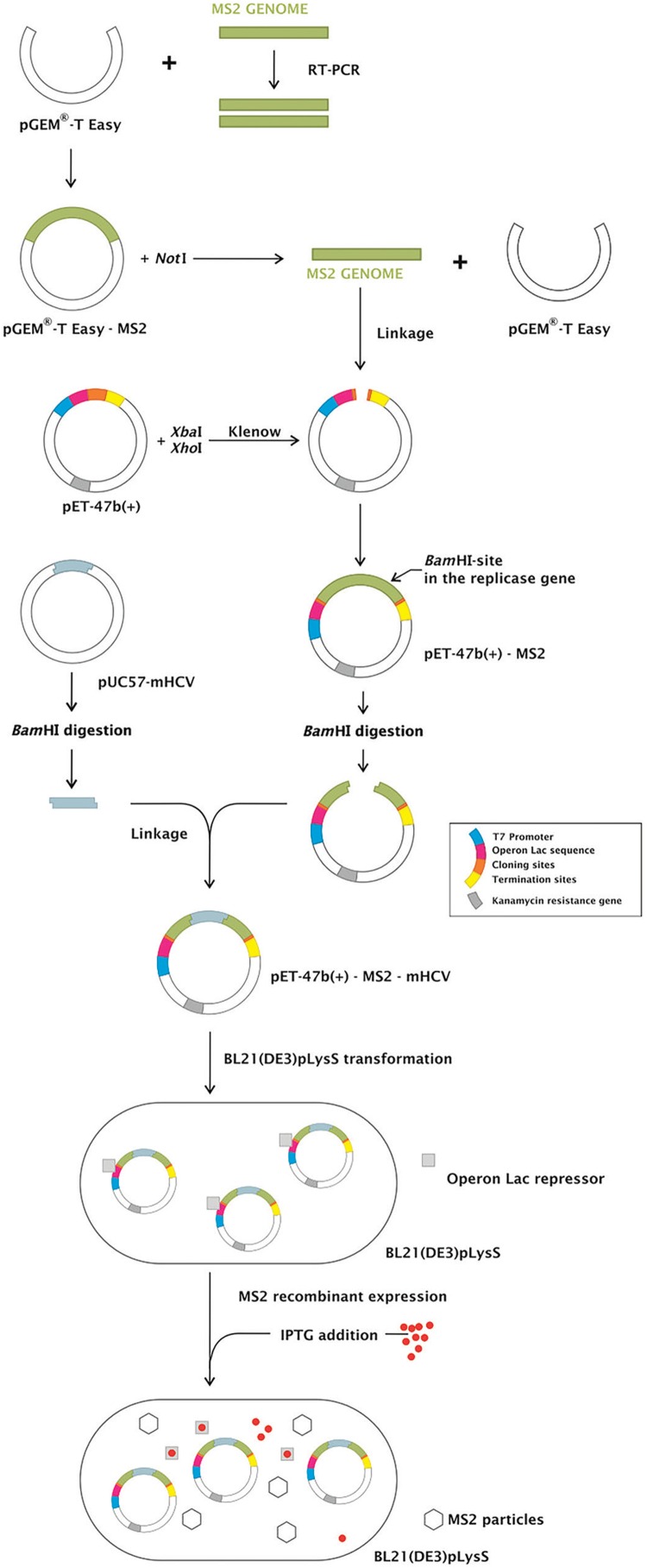
: strategy to MS2-like and MS2-modified hepatitis C virus (MS2-mHCV) internal control (IC) construction. MS2 genome was inserted in the pGEM-T Easy plasmid. After the digestion with *Not*I, it was removed from the pGEM-T Easy and ligated into pET-47b(+) generating pET-47b(+)-MS2. The mHCV sequence was inserted into the pET-47b(+)-MS2 in the MS2 replicase gene at the *Bam*HI site, generating pET-47b(+)-MS2-mHCV. The constructed expression vectors pET-47b(+)-MS2 and pET-47b(+)-MS2-mHCV transformed in BL21(DE3)pLysS were used to produce MS2-like and MS2-mHCV IC, respectively, represented as MS2 particles.

*Internal control construction* - We developed a competitive internal control to be used in assays to detect the nucleic acid from HCV, based on the MS2 bacteriophage. This IC could also be used as non-competitive control in any RNA virus detection test. The MS2 bacteriophage genome was inserted in pET47b(+) plasmid, generating pET47b(+)-MS2. pET47b(+)-MS2-mHCV was constructed after the insertion of a mutated HCV sequence into a unique *Bam*HI site (within the replicase gene of MS2 genome) of pET47b(+)-MS2. All the constructs were confirmed by sequencing. An overview of the strategy is shown in Fig. 2.

Both pET47b(+)-MS2 and pET47b(+)-MS2-mHCV could generate MS2-like particles through the synthesis of MS2 genome by T7 RNA polymerase. pET47b(+)-MS2-mHCV generated particles whose genome harbored 144 nt of a HCV sequence in the middle of the replicase gene, and as consequence a non-functional enzyme was produced, blocking replication of the genome. From now on, these mutant bacteriophage particles will be referred to as MS2-like IC and MS2-mHCV IC, respectively.

**Fig. 3:**
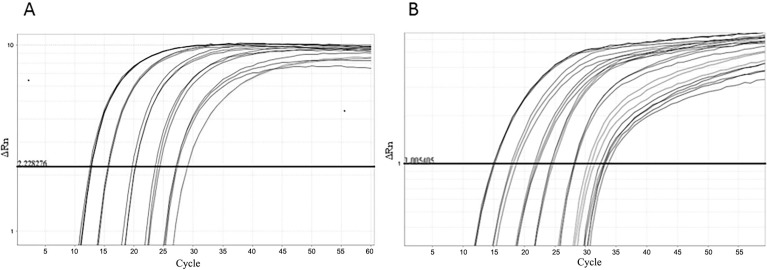
: internal control (IC) amplification. Real-time polymerase chain reaction (PCR) plots of MS2-like IC (A) and MS2-modified hepatitis C virus (mHCV) IC (B) in one-step real-time reverse transcriptase (RT)-PCR The samples consist of 10-fold dilutions, with the more concentrated sample having 7.5 × 104 PFU/mL and 2.1 × 105 PFU-eq/mL for MS2-like and MS2-mHCV IC, respectively. The horizontal line corresponds to the measured fluorescence.

One step real-time reverse transcriptase-PCR (RT-PCR) was developed and optimised for each control. The RNA was extracted from ten-fold dilutions of MS2-like and MS2-mHCV particles stocks. The nucleic acid from MS2-like IC was amplified using primers MS2-F and MS2-R and probe MS2-P (Fig. 3A). The mean PCR efficiency was 92%. The RNA from MS2-mHCV particles was amplified using primers HCV-F and HCV-R1 and the probe mHCV-P (Fig. 3B). This reaction had a mean efficiency of 104%. Despite the amplification of several quantities being carried out over eight logs, the test linearity comprised six logs for both ICs.

MS2-mHCV IC could not be quantified by plating assays, since it invades but does not replicate and lyse the bacterial cell; therefore, we decided to quantify it by real-time PCR compared with the MS2-like curves previously quantified. RNA was extracted from three samples of MS2-mHCV IC and subjected to real-time PCR with primers MS2-F and MS2-R and the probe MS2-P, along with the standard curve that comprised 10-fold serial dilutions of MS2-like RNA. The MS2-like was titled by plating assays with 7,5 x 10^4^ PFU/mL and the MS2-mHCV IC with 2,1 x 10^5^ PFU-eq.

*Non-competitive internal control for the HCV detection reaction* - The utility of MS2-like particles as a non-competitive ICs was evaluated in a one-step format multiplex real-time RT-PCR for HCV detection. The use of a set of primers and probes for amplification of the MS2 genome makes these MS2-like particles useful as ICs in assays for any RNA virus.

The HCV primers were designed to amplify the region between positions 130 and 201 (GenBank reference sequence AF009606.1), which is conserved among the different genotypes (Duarte et al. 2010). The MS2-like particles were tested as a non-competitive internal control with the same primer pair and probe used in the singleplex reaction, plus the primers and probe to detect HCV (Table I).

**Fig. 4:**
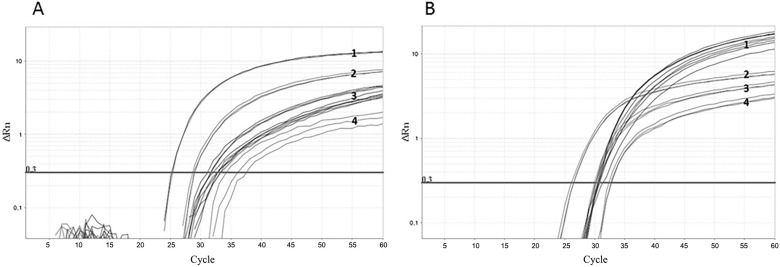
: amplification of hepatitis C virus/internal control (HCV/IC) multiplex reactions. (A) HCV sample (3) spiked with 1 (1), 10-1 (2) and 10-2 (4) PFU MS2-like IC. (B) HCV sample (1) spiked with 1 (2), 10-1 (3) and 10-2 (4) PFU -eq MS2-mHCV IC. The horizontal line corresponds to the measured fluorescence.

The sample PHW804-01 from the HCV RNA Linearity Panel was diluted to 8.25 x 10^5^ UI/mL and was spiked with 5 mL MS2-like IC (1, 10^-1^, 10^-2^ PFU). The RNA from HCV and MS2-like IC was extracted and amplified in a multiplex real-time PCR (Fig. 4A). The HCV amplification was detected at C_T_s 31 to 33, whereas the MS2-like was in C_T_s 26, 29 and 34 to 37 (average 35). The experiments indicated that the IC amplification was reproducible, with negligible interference in HCV detection, independently of the amount of MS2-like IC. Therefore, we opted to use 1.0 × 10^-1^ PFU of MS2-like IC in subsequent tests.

*Competitive IC for the HCV detection reaction* - The MS2-mHCV was evaluated as a competitive IC in a one-step real-time PCR for HCV detection.

The same HCV region targeted for HCV detection was inserted into MS2-mHCV with some mutated bases in relation to the wild-type (WT) sequence. The multiplex PCR required only one HCV primer pair and two probes to discriminate between the WT and the mutated sequence. The probes for HCV or MS2-mHCV IC were designed against positions 149 to 169 of the reference sequence (Table I).

PHW804-01 at 8.25 x 10^5^ UI/mL was used in the multiplex reaction with 1, 10^-1^, 10^-2^ PFU-eq of MS2-mHCV IC (Fig. 4B). All quantities of MS2-mHCV IC tested had a suitable amplification curve (C_T_ 28 to 34), despite the greater variability of 10^-2^ PFU-eq amplification. The multiplex reaction with the different amounts of competitive IC of MS2-mHCV did not affect the amplification of HCV. In subsequent tests, we added 10^-2^ PFU-eq of MS2-mHCV IC to each sample.

*Comparison between competitive and non-competitive ICs in HCV detection* - Fig. 4 shows that the HCV C_T_ values in the multiplex reaction were very close, regardless of whether the IC was competitive or non-competitive. The evaluation of the multiplex HCV/IC real-time PCR was extended by adding MS2-like IC (10^-1^ PFU) and MS2-mHCV IC (10^-2^ PFU-eq) to HCV Panel samples with 2.5 × 10^6^, 2.5 × 10^5^, 2.5 × 10^4^, 2.5 × 10^3^, 2.5 × 10^2^ and 2.5 × 10 UI/mL. MS2-mHCV IC were detected in all samples at a mean C_T_ of 30.9 The mean C_T_ for MS2-like IC was 26.7. From 2.5 x 106 to 2.5 x 10^3^, the HCV amplification curves were similar among the reactions using the competitive and non-competitive IC. These values are reported in Table II and the plots of the amplification images are shown in Supplementary data, Figure.

**TABLE II:** Real-time polymerase chain reaction results of the hepatitis C virus-internal control (HCV-IC) multiplex reaction

Sample (UI/mL)	HCV/MS2-like		HCV/MS2-mHCV	
	
	CT^*^ HCV	CT IC	CT HCV	CT IC
2,500,000	23.1	26.3	25.1	30.4
250,000	26.2	26.5	27.5	30.8
25,000	29.6	26.9	30.4	30.9
2,500	39.5	27.2	37.3	30.8
250	45.0	27.2	39.3	30.9
25	NA	26.3	NA	31.8

^*^: CT = cycle threshold; NA: not amplified.

*Non-competitive IC for the human immunodeficiency virus-1 (HIV) detection reaction* - In addition, the MS2-like particles were evaluated as a non-competitive IC in a one-step format multiplex real-time PCR for HIV detection, demonstrating that these controls can be used in assays for other RNA virus. Primers and the probe to amplify the IC were the same as in the HCV/MS2-like multiplex reaction.

**Fig. 5:**
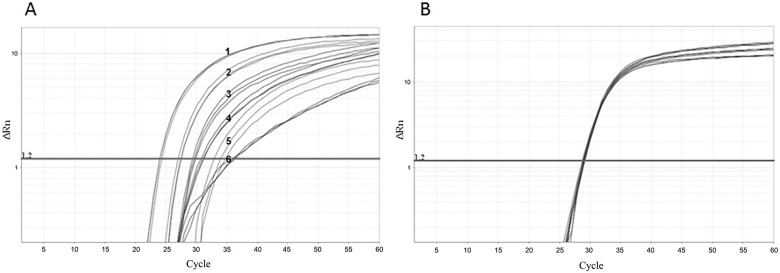
: amplification of human immunodeficiency virus/internal control (HIV/IC) multiplex reactions. (A) HIV sample (3, 4 and 6) spiked with 1 (1), 10-1 (2) and 10-2 (5) PFU of MS2-like IC. Using 1 PFU of MS2-like IC (1), the HIV CT was 36 (6); using 10-1 PFU (2) the HIV CT was 30 (4); and using 10-2 PFU (5) the HIV CT was 29 (3). (B) HIV in a singleplex real-time reverse transcriptase polymerase chain reaction (RT-PCR) reaction. The horizontal line corresponds to the measured fluorescence.

One HIV clinical sample with a viral load of log 4 was spiked with 5 mL MS2-like IC (1, 10^-1^, 10^-2^ PFU). The RNA from HIV and MS2-like IC was extracted and amplified in a multiplex real-time PCR (Fig. 5). The HIV amplification was detected at C_T_s 29 to 36, while the MS2-like in C_T_s 24, 27 and 33. The results showed that 1 and 10^-1^ PFU disturbed HIV amplification and 10^-2^ PFU appeared to be the optimum amount of control for this reaction, because the resulting C_T_ of HIV was equal to that in the test without the control.

## DISCUSSION

The aim of the present work was to construct an IC to assess the different steps of an RNA virus detection test based on real-time PCR, from RNA extraction to the amplification reaction. For that, MS2 was chosen as a suitable virus to be added to the samples. MS2 is an RNA virus that is encapsulated such that the genome is protected from RNases, it is easily handled with basic molecular biology techniques, and is non-pathogenic to humans, requiring no extra care regarding biological safety in addition to those necessary to work with *E. coli* and with human blood samples. HCV, an RNA virus, was chosen as a model for the construction and validation of this control.

MS2 bacteriophage belongs to *Leviviridae* family and its 3,569 nt single strand RNA genome encodes four proteins: (i) the maturase, involved in the infection through the *E. coli pilus*; (ii) the coat protein, which self-assembles in an icosaedric shape formed by 180 monomers; (iii) the lysis protein, involved in the bacterial cell lysis; and (iv) the replicase protein, an RNA-dependent RNA polymerase (RdRP)(Fiers et al. 1976).

To construct the IC, the whole MS2 genome was cloned into vector pET47b(+)(Novagen), generating the first version of the IC. This plasmid transcribes an MS2 transcript via T7 RNA polymerase and is capable of producing MS2 particles. These MS2 particles can be used as a non-competitive IC in PCR reactions to detect RNA viruses, using an oligonucleotide set specific to the MS2 genome.

To transform this non-competitive IC into a competitive IC for an RNA virus reaction, a modified sequence of the elected virus must be inserted into the MS2 genome. This modification in the sequence is necessary to allow a differential probe to discriminate the pathogen sequence from the IC in the same plate well. Thus, the second version of our IC, the competitive IC, was generated by inserting a modified HCV sequence in the middle of the MS2 replicase gene. The inserted sequence was identical to the wild-type sequence in length and GC content, hence the amplification efficiencies of the HCV and the control were the same. Any factor interfering in one target amplification would interfere with the other at the same rate.

The introduction of this sequence into the replicase gene produced a non-functional RNA polymerase. This meant that once transformed, the bacteria produced viral particles via the plasmid (through the T7 polymerase encoded in the bacterial genome). These particles, if by any chance infected by other non-T7 polymerase expressing bacterial stock, could not replicate. A fortuitous infection by a T7 polymerase-expressing strains would not generate phage particles without induction. A nucleic acid test (NAT) for HCV and HIV was developed by Fiocruz/Brazil to be used in Brazilian blood banks (Patent number PI0600715-5/INPI/Brazil). We chose to test the performance of our ICs (competitive and non-competitive) using the same primers and probes used in this test. The applicability of the competitive and non-competitive ICs for HCV detection was demonstrated in this work. We also showed the applicability of the non-competitive control to HIV-1 detection by real-time PCR, although the reaction should be optimised to establish an ideal concentration of the IC that does not interfere with HIV-1 amplification, as has been done for HCV. We showed the results for one-step real-time RT-PCR, but the real-time PCR for HCV using our ICs were also successful and reproducible in a two-step format (data not shown).

Previous works demonstrated the use of wild-type MS2 particles as ICs for several pathogens (Rolfe et al. 2007). Dreier and coworkers used MS2 as a control for HCV diagnosis (Dreier et al. 2005). In these studies, however, MS2 was produced via the biological cycle (infection and lysis of bacteria) and we produced MS2 particles from a plasmid, in a regulated fashion, diminishing the steps that could cause MS2 to be a contaminant in laboratory-stored the bacterial stocks (Villanova et al. 2007). Another advantage of using a plasmid containing the MS2 sequence is that the replication of the plasmid via the bacterial DNA polymerase generates fewer mutations than the MS2 replication by its RNA polymerase (replicase) (Fiers et al. 1976). The avoidance of the risk of contamination of laboratory bacterial stocks and the non-generation of mutations by replication through RdRP are advantages of the pseudoviral particles use and our competitive IC satisfied those requirements (Pasloske et al. 1998).

One of our concerns was the possibility of IC interference in the detection limit of the test. Fiocruz’s test has a sensitivity of 300 IU/mL for HCV. Albertoni et al. (2010) reported a limit of detection for an one-step RT-PCR for HCV detection as 310 IU/mL. Wendel et al. (2007) reported a detection limit of 500 IU/mL for their in-house HCV real-time RT-PCR test. Those two works did not use any IC. Villanova et al. (2007) developed a competitive control for HCV diagnostics based on another virus, Qβ, and obtained a detection limit 500 IU/mL. The work of Dreier et al. (2005), who performed an HCV test with MS2 as an IC, was able to detect 100 IU/mL, although in the duplex reaction a decrease in HCV fluorescence was observed when compared to the singleplex reaction of 100 IU/mL. Our system was able to detect 250 IU/mL, a value that is comparable with Fiocruz’s test. Thus, the addition of the IC to an already optimised reaction is possible without compromising the original performance, after a few steps of optimisation.

Pseudovirus commercial controls also based on MS2 particle are also available in several formats for several pathogens, including HCV. Their costs, however, hamper its broad use in developing countries. Our IC approach can be used in these countries, since it can be developed in-house. Any sequence of interest can be inserted at the *Bam*HI site in the framework of the plasmid containing the MS2 sequence which provides versatility to create non-competitive or competitive ICs.

An attractive feature of this system is that it allows the construction of a multicontrol by the insertion of sequences from more than one pathogen, increasing its applicability as an IC for diagnosing different RNA viruses.

In this work we constructed ICs to RNA virus diagnosis by real-time RT-PCR, using HCV as a model. Despite the need to extend their evaluation using a larger characterised panel of clinical samples, we demonstrated that both competitive and non-competitive ICs successfully assessed the HCV amplification performance without compromising the detection of low titers samples.
