# Supplementary material for: Internal control for real-time polymerase chain reaction based on MS2 bacteriophage for RNA viruses diagnostics
Source: Mem Inst Oswaldo Cruz. 2017 Apr 6;112(5):339–47. doi: 10.1590/0074-02760160380 (PMC5398160; doi:10.1590/0074-02760160380)
Supplement: Supplementary file 1 [file 0074-0276-mioc-0074-02760160380-suppl01.pdf]

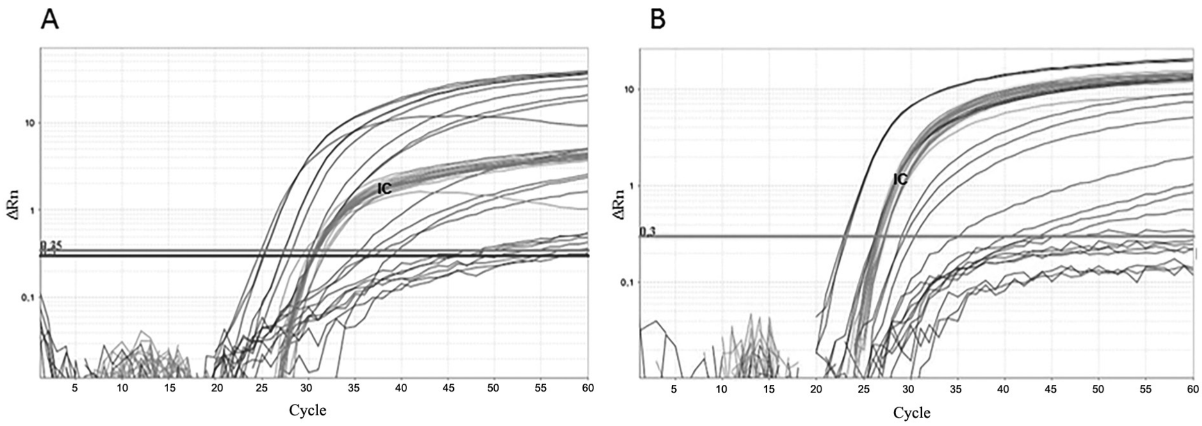


Amplification of hepatitis C virus/internal control (HCV/IC) multiplex reactions. HCV panel samples with $2.5 \times 10^6$, $2.5 \times 10^5$, $2.5 \times 10^4$, $2.5 \times 10^3$, $2.5 \times 10^2$ and $2.5 \times 10^1$ UI/mL spiked with 5 µL IC: $1 \times 10^{-1}$ PFU of MS2-like IC (A) or $1 \times 10^{-2}$ PFU-eq of MS2-mHCV IC (B). Curves corresponding to IC amplification are labeled as IC and the remainder represents the amplification of different amounts of HCV particles. The horizontal line corresponds to the measured fluorescence.
